# Mixed methodology in human brain research: integrating MRI and histology

**DOI:** 10.1007/s00429-023-02675-2

**Published:** 2023-06-26

**Authors:** Anneke Alkemade, Rosa Großmann, Pierre-Louis Bazin, Birte U. Forstmann

**Affiliations:** 1 Integrative Model-Based Cognitive Neuroscience Unit, Department of Psychology, University of Amsterdam, Amsterdam, The Netherlands; 2 Department of Neurophysics, Max Planck Institute for Human Cognitive and Brain Sciences, Leipzig, Germany; 3 Department of Neurology, Max Planck Institute for Human Cognitive and Brain Sciences, Leipzig, Germany

**Keywords:** Postmortem, MRI, Histology, Reconstructions, Co-registration

## Abstract

Postmortem magnetic resonance imaging (MRI) can provide a bridge between histological observations and the in vivo anatomy of the human brain. Approaches aimed at the co-registration of data derived from the two techniques are gaining interest. Optimal integration of the two research fields requires detailed knowledge of the tissue property requirements for individual research techniques, as well as a detailed understanding of the consequences of tissue fixation steps on the imaging quality outcomes for both MRI and histology. Here, we provide an overview of existing studies that bridge between state-of-the-art imaging modalities, and discuss the background knowledge incorporated into the design, execution and interpretation of postmortem studies. A subset of the discussed challenges transfer to animal studies as well. This insight can contribute to furthering our understanding of the normal and diseased human brain, and to facilitate discussions between researchers from the individual disciplines.

## Introduction

**Fig. 1:**
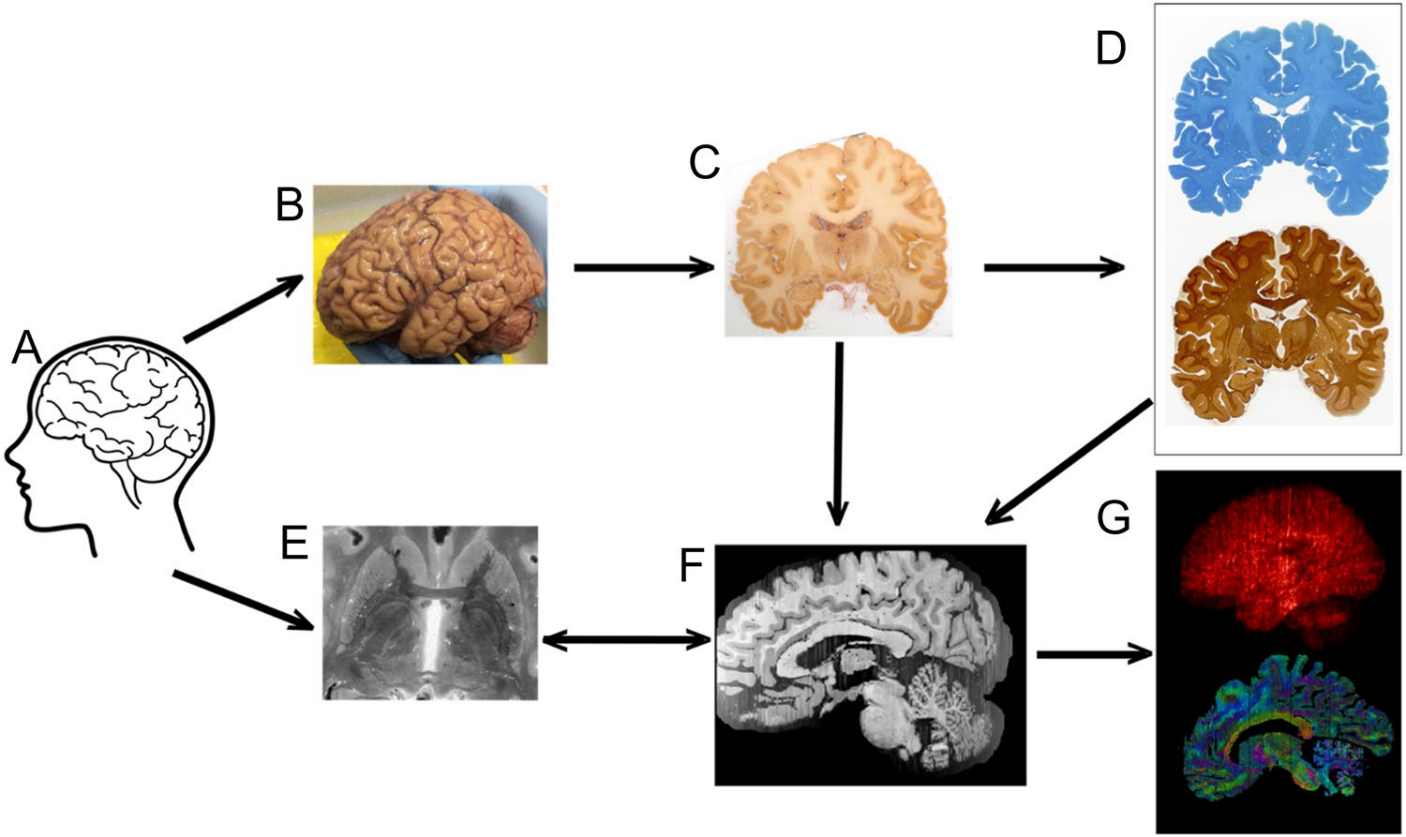
Combining MRI and histological data. Tissue (**A**) is subjected to MRI scanning after MRI has been performed. Brain autopsy is performed (**B**) followed by blockface imaging (**C**) and (immuno)histochemical staining (**D**). 3D reconstructions are combined with MRI data (**E**) in reconstructed blockface space (**F**), after which imaging parameters can be derived (**G**). Adapted from Alkemade et al. (2020)

Grasping the full complexity of the structure and function of the human brain cannot be achieved using a single research modality. Today’s knowledge results from incremental data obtained through a broad spectrum of research approaches, ranging from the extrapolation of findings from a variety of non-human species, to data obtained through a plethora of brain imaging techniques applied in humans. The most commonly used approach to image the whole human brain in vivo with the purpose of studying neuroanatomy and function is magnetic resonance imaging (MRI). MRI techniques are continuously undergoing technological developments aimed to further improve hardware, MR sequences as well as algorithms used for image reconstructions. An approach that is gaining popularity within the field of structural MRI is the investigation of human postmortem specimens. In an increasing number of studies, detailed MRI is followed by histological processing of the tissue. The integration of the methodologies when combining MRI and histology requires the balancing of the opportunities and challenges associated with the individual techniques, optimization choices may differ from choices made if only histological or MRI techniques are uses. This overview provides insight in the choices that are made. In spite of the compromises, the combined approach is stirring excitement in the field, since the spatial alignment of MRI and histological reconstructions bring forward new opportunities that allow fine-grained anatomical validation of MRI results, and more detailed studies on the 3D structure of the brain (Fig. 1). The acquired level of anatomical detail cannot be achieved using MRI techniques in vivo. Vice versa, translation of histological results to MRI observations opens new possibilities to determine the generalizability of older histological findings which were based on a small number of specimens.

The scientific value of pushing the signal-to-noise ratio and spatial resolution of (quantitative) MRI to further improve anatomical contrast is recognized by the research community, and the same holds for efforts moving histology from a 2D to a 3D field. Building a bridge between modalities gives rise to synergistic research that allows the creation of unified maps of the human brain registered to a common template space (Alkemade et al. 2022). Such datasets provide valuable resources for further research.

Over the years, several groups have dedicated their time and effort to develop methodologies that allows the combination microscopy and MRI approaches. Their scientific resourcefulness has led to the development of various pipelines to acquire multimodal data within specimens, either through histological processing tissue blocks or whole brains, with or without spatial interpolation (Chakravarty et al. 2006; Yang et al. 2013; Alegro et al. 2016; Alho et al. 2017, 2018; Mollink et al. 2017; Sitek et al. 2019). In Table 1, we provide an overview of studies presenting research approaches that apply postmortem MRI in humans, combined with histological tissue processing. The identified studies integrate histology and histochemistry with ultra-high field MRI observations, closing the gap between imaging modalities, and illustrating developments in the field. Table 1 does not provide an exhaustive overview nor a comparison between studies. It highlights important efforts combining MRI data with histological sections and co-registrations, and illustrating the diversity of the developed approaches. We omitted efforts that applied MRI validation by means of visual inspection of microscopy sections without registration, and efforts matching histological and MRI images of pathological masses in the brain. In addition, we focus on ultra-high field MRI combined with histological reconstructions. This choice was made to emphasize the possibilities of ultra-high field MRI imaging in postmortem tissues, although we would like to acknowledge that high-quality images can also be obtained at 3 Tesla (T). A direct comparison between different datasets listed in Table 1 is challenging due to substantial variation in MRI methodology, histological tissue processing approaches, as well as donor characteristics and lies beyond the scope of the current discussion. In addition, we would like to point out that many of the considerations discussed are focused on the study of human brain material, but translate to studies on other species as well.

**Table 1:** Studies co-registering ultra-high-field MRI and histological data

	Type of donor	Brain area	Number of specimens	Study details
Amunts et al. (2005)	NDC	Hippocampus, the amygdala and the entorhinal cortex	10	Reconstructions provide partial histological coverage, registrations to MRI space are performed within subjects, probabilistic maps are available for reuse
Chakravarty et al. (2006)	NDC	Basal ganglia and thalamus	1	Reconstructions provide partial histological coverage based on archival data. No registration to MRI space is performed within subjects. Data are co-registered to MRI template space
Yelnik et al. (2007)	NDC	Basal ganglia	1 (selected from 8 scanned/sectioned specimens)	Reconstructions provide partial histological coverage, registrations to MRI space is performed within subjects, and resulting maps are available for reuse
Augustinack et al. (2010)	NDC	Medial temporal lobe	6	Reconstructions provide partial histological coverage, registrations to MRI space are performed within subjects
Krauth et al. (2010)	NDC	Thalamus	6 (3 with MRI)	Maps are created based on histological data, which are partially registered to within subject MRI data. Maps created are available on request
Kolasinski et al. (2012)	MS	Thalamus/whole brain	9	Histological specimens were analyzed using (semi)quantitative approaches, and compared to MRI parameters within subjects. Co-registration of the data was not reported
Yang et al. (2013)	N/A	Whole brain	2	Reconstructions provide partial histological coverage, registrations to MRI space is performed within subjects
Morel et al. (2013)	NDC	Focus on insula	4	Reconstructions provide partial histological coverage, registrations to MRI space is performed within subjects
Kujovic et al. (2013)	NDC	Dorsal extrastriate cortex	10	Reconstructions provide partial histological coverage, registrations to MRI space is performed within subjects, and resulting maps are available for reuse
Adler et al. (2014)	NDC	Hippocampal formation	1	Reconstructions provide partial histological coverage, registrations to MRI space is performed within subjects
Seehaus et al. (2015)	NDC	Large part of the left hemisphere	1	Information derived from the histological samples is co-registered with the MRI data of the same specimen
Mai et al. (2015)	NDC	Whole brain	3	Histological data are registered to MNI template space. Resulting maps are available for reuse
Ding et al. (2016)	NDC	Whole hemisphere/brain	1	Individual histological sections are matched with the corresponding to MRI levels of the same subjects. Histological data, MRI data and resulting maps are available for reuse
Iglesias et al. (2018)	NDC	Thalamus	6	Reconstructions provide partial histological coverage, registrations to MRI space is performed within subjects, and are co-registered with atlas data. Resulting maps are available for reuse
Lebenberg et al. (2018)	NDC	Subcortical auditory system	1 brain stem	Presented data are reused from existing databases, and registered to MRI template space. Derived maps are available for reuse
Sitek et al. (2019)	NDC	Thalamus and brainstem	1	Connectivity maps of the auditory system are coaligned with BigBrain and registered to MNI space. The 7 T MRI contrasts are available for reuse
Huszar et al. (2019)	Motor neuron disease NDC	Staged dissection	13 MND, 3 NDC	Histology coordinates are registered to MRI images within the same specimens. The resulting Tensor Image Registration Library is available for reuse
Roseborough et al. (2020)	AD Cardiovascular disease	Selected sections	20 (5 NDC AD, CVD, 5 AD& CVD)	Individual histological sections are mapped to MRI space within the same specimens. Based on 19 histological sections
Ushizima et al. (2022)	AD	Whole brain	2	Patch registration is performed within the same specimens
Alkemade et al. (2022)	NDC	Whole brain	2	Whole-brain histology is 3D reconstructed and co-registered with 7 T quantitative MRI. Histological data, as well as qMRI are available for reuse

*AD* Alzheimer’s disease, *CVD* cardiovascular disease, *MNI* Montreal Neurological Institute, *MS* multiple sclerosis, *NDC* non-demented control We searched PubMed and GoogleScholar using the keywords “post mortem”, “mri” and “histology”, and subsequently repeated the search after adding the keywords “ex vivo” and “post-mortem”. In addition, “atlas” and “MNI” were used to find publications aimed at atlas construction and the registrations to Montreal Neurological Institute (MNI) standard space

Only datasets obtained with 7 T or higher field strength MRI are listed

The research presented in Table 1 reflects an impressive amount of manual labor involved in histological processing, with a substantial amount of data being made available for reuse by other research groups. The majority of these efforts concern smaller or larger tissue blocks, and not whole-brain samples. In total, 4 human postmortem whole brains have been sectioned, fully processed for (immuno)histochemical staining, and subsequently 3D reconstructed (Amunts et al. 2013, 2020; Alkemade et al. 2022). Of one of these datasets, the derived atlases are available for reuse (Amunts et al. 2020). The other three (raw) datasets are shared for reuse by the scientific community (Amunts et al. 2013; Alkemade et al. 2022). The BigBrain dataset includes over 7400 20 µm microscopy sections using a single staining Nissl-like procedure but does not include high-quality MRI data (Amunts et al. 2013). To our knowledge, so far only our own efforts provide whole-brain data and reconstructions of 200 µm sections labeled using 5 different (immuno)histochemical procedures, reconstructed in a common blockface space together with 7 T quantitative MRI at a 200 µm isotropic resolution (Alkemade et al. 2022). To allow reuse of these and other histological datasets, the data needs to be accessible, and adequately annotated. Ideally, researchers reusing shared data can derive all relevant information from the publication that accompanies the data. However, each individual research field has its own intricacies. The implicit knowledge present in a research field contributes to shaping the research design, and the results of choices based on such implicit knowledge are automatically transferred into the datasets. The implicit information is not necessarily available to researchers from other fields that reuse the data from these studies. Therefore, we now highlight important considerations for the specific design of studies that combine MRI and histology. We chose to tailor the discussion towards the study of the human brain, which is special given the number of factors that are beyond the control of the researchers such as advanced aging, disease parameters, pharmacological treatment, and long postmortem intervals. We would like to acknowledge that a proportion of the challenges transfer to the combination of MRI and histology in animal studies as well.

The provided background knowledge will not only facilitate the interpretation of combined MRI and histological studies available in literature, it can also serve as a starting point for research groups looking to venture into the field of combined MRI and histology.

We hope that this overview will facilitate discussion between researchers from the adjoining research field. For example researchers working in either the field of MRI, image analysis, *or* histology, will benefit from insight in the compromises are made in the experimental setup to accommodate mixed methodology. Finally, the discussions are of interest to researchers reusing existing datasets that combine MRI and histological data.

## The practice of combining MRI and histology

The combination of MRI and histology allows a detailed validation of the MRI images. MRI contrasts are used to visualize the underlying neuroanatomy, but are sensitive to susceptibility artifacts that can result in geometric distortion and signal dropout in images, contributing to distorted representations of the underlying the anatomy (Lüdeke et al. 1985; Chang and Fitzpatrick 1992). The impact on the visualization is difficult to assess. In addition, inferences have been made linking MRI contrast and anatomical features, which are subsequently supported by histological assessments (Sun et al. 2015). In some cases, the validation of MRI contrasts with microscopy studies has led to reassessment of the inferred link between structure and contrast (Brammerloh et al. 2022). MRI and microscopy approaches thus provide different levels of insight into the anatomy of the human brain.

3D reconstructions of human brain tissue using microscopy slides can provide a submillimeter level of anatomical description including information on the molecular fingerprint of individual brain areas (Makris et al. 2013; Mai et al. 2015; Iglesias et al. 2015, 2018; Alkemade et al. 2019, 2022; Amunts et al. 2020). Acquiring data at a spatial resolution that allows the visualization of individual neurons of the human cerebrum is beyond the reach of available state-of-the-art MRI techniques. These studies provide detailed insight in the anatomy of the human brain, but it is unknown to what extent postmortem observations are representative of the average healthy human brain. These questions cannot be answered using postmortem studies in view of inevitable effects of the process of dying which are likely to be transferred to the postmortem anatomy of the brain. In addition, postmortem studies additionally suffer from an inherently low number of observations due to their labor-intensive nature, and the limited availability of well-documented high-quality donor brains.

The increased attention for a whole-brain postmortem MRI approach is fueled by the possibilities for obtaining higher spatial resolutions as compared to in vivo scans, in combination with long scan periods, using either clinical or customized tissue coils (Miller et al. 2011; Plantinga et al. 2016; Edlow et al. 2019; Fritz et al. 2019; Alkemade et al. 2020). The data derived from postmortem specimens using submillimeter 7 T or higher field MRI is often very detailed with an exceptional contrast-to-noise ratio (CNR). In addition, the combination of MRI and histology creates a framework that allows the translation between postmortem and in vivo findings through the co-registration of the data from the respective research fields in MRI standard templates, such as the MNI 2009B and Colin-27 Brain (Holmes et al. 1998; Fonov et al. 2011). In practice, these studies present a compromise in which MRI and histology requirements need to be balanced. Like with any other research technique for optimal research design, background knowledge on the strengths and limitations is crucial, as well as methodology to recognize and mitigate potential limitations.

The study of the human brain using postmortem brain specimens using MRI and histology includes coping with tissue characteristics and their alterations due to stunned physiology, tissue degradation, fixation procedures and concessions that need to be made to accommodate both MRI and histological research. Some elements of the protocols used in histology are common practice and can be tailored to create a better bridging across fields. In addition, the histological protocols can be tailored to the needs of researchers performing image reconstruction, and co-registration of histological images with MRI data. This can for instance include the histological staining of tissue characteristics resulting in the visualization of mutual information shared with MRI contrasts (e.g., myelin). An open discussion between researchers, sharing the background knowledge, understanding the limitations and workload involved, will help to bridge modalities. When developing an integrated research protocol, the leading question should be the research question at hand and both disciplines should be open to adapting their operationalized protocols, and adjusting their standards in service of the bridging to the other field. In practice, this means that researchers from the field of microscopy may not be able to study the anatomy at the level of detail to which they are accustomed to, since only very few groups in the world have the resources to achieve these levels of detail, and subsequently creating 3D reconstructions of the whole brain, or tissue blocks. At the same time, researchers from the field of MRI will need to accommodate changes in MRI characteristics resulting from steps required to prevent tissue decay that would interfere with histological processing. In addition, the time required to process and analyze large human brain specimens hampers the rapid publication of studies applying state-of-the-art MRI techniques.

## Comparisons between living and dead brains

Postmortem studies come with inherent unknowns on the translatability of the observations to the in vivo situation. Important questions that immediately rise are: is the postmortem brain still (partially) representative of the living brain? What does postmortem MRI tell us about the in vivo situation? What non-MRI related factors differ between in vivo and postmortem scans? What factors can and cannot be controlled for in postmortem studies?

## A direct comparison between postmortem and in vivo MRI

A straightforward approach to determine to what extent the human postmortem brain is representative of the living brain is to perform a within subject study providing a direct comparison between the in vivo and postmortem state. Although this approach has the potential to answer a crucial question, setting up such a study is ethically delicate, and this may explain the largely absent data in scientific literature. We are aware of only a single unique case report that provides a direct, within subject comparison of ante- and postmortem MRI before tissue fixation. A 37-year-old male patient with familial early-onset Alzheimer’s disease was scanned 4 days ante- and 9 h postmortem using the same imaging setup and protocol allowing direct comparisons (Boon et al. 2019). Despite the limitation that this case report does not concern a healthy brain nor does it allow statistical between group comparisons, it does provide a valuable insight in the potential alterations in brain imaging differences in vivo and postmortem. A number of clear differences were observed before and after death. The susceptibility weighted images obtained postmortem clearly reflected deoxygenation and blood stasis as compared to the in vivo scans. In addition, as expected, diffusivity was drastically decreased after death (50–60%), whereas brain volume and fractional anisotropy were increased. Whether these changes reflect a combination of vasogenic and cytotoxic edema could not be assessed. Importantly, the observed volume increase was not homogenous across the brain, which has implications for the interpretation of postmortem studies in general. In addition to the clear difference in MRI characteristics as well as direct effects of death on brain structure, there were many similarities between the ante- and postmortem scan. Cortical thickness measured before and after death were strongly correlated, and both in vivo and postmortem cortical thickness measurements showed the same shape distribution in relation to the histopathology, which indicates that these observations are tightly linked. This case report provides a rare insight into the alterations resulting from stunned physiology, without allowing to tease apart the individual factors that contributing to the differences observed between these observations. Furthermore, this unique case study shows that these efforts are within reach in clinical cohorts, although they require a long scientific dedication resulting from the prospective data collection which is crucial to this work. The creation of research cohorts such as the Normal Aging Brain collection Amsterdam (NABCA) (Jonkman et al. 2019), of patients who are followed during life, and donate their clinical data and their brain after death represent a potential wealth of scientific data.

## Comparison across individuals

When within subject observations are not available, which is in the large majority of cases, an alternative approach is the co-registration of individual postmortem observations to standard MRI templates. The anatomy and MRI characteristics of the postmortem human brain may have been altered, but despite these alterations, a satisfactory registration between postmortem brain specimens, as well as the reconstructed BigBrain (Xiao et al. 2019) and blockface reconstructions (Alkemade et al. 2020a, 2022) can be achieved. Validation of the registration can be performed through the determination of overlap between the size and location of individual structures that are visible on both in vivo as well as on postmortem MRI contrasts. This approach can be used to create an important bridge between postmortem and in vivo datasets. These co-registrations are particularly interesting for the determination of the expected location of individual brain structures for the matching of brain activation patterns such as those obtained in fMRI studies to individual brain nuclei that are challenging to identify on in vivo MRI scans (Forstmann et al. 2017). In addition, these co-registrations can serve as a bridge to translate between microscopy reconstructions and MRI through the availability of mutual information between the MRI modalities, which allow the use of available MRI tools that are used for co-registration of MRI data in vivo.

## Factors causing differences between in vivo and postmortem observations

### Antemortem and perimortem factors

Although the gross anatomy of the brain does not change as a result of death, cohorts of brain donors differ substantially from cohorts commonly included in in vivo MRI studies in multiple ways. Brain donors are generally older than the age in convenience cohorts included in in vivo MRI studies, which often consist of young undergraduate students. Brain characteristics such as iron accumulation and myelin content change as a result of normal aging effects are expected to contribute to differences with in vivo MRI studies. It is clear from the literature that brain (MRI) characteristics differ between age groups (Hallgren and Sourander 1958; Zecca et al. 2004; Raz and Rodrigue 2006; Shen et al. 2008; Daugherty and Raz 2013; Miletić et al. 2022). Since human brain specimens are not readily available, interpretation of potential effects of age between postmortem specimens and in vivo observations are more easily achieved through validation/extension of the in vivo studies through the inclusion of older age groups. This approach, however, cannot be easily transferred to other potential confounders such as terminal disease, co-morbidities, disease duration, pharmacological treatment and cause of death (e.g., Alkemade et al. 2005, 2012; Weiss et al. 2015; ten Kulve et al. 2016). Participants in the control group in in vivo studies generally do not have clinically overt disease, since somatic disease is often defined as an exclusion criterion. In postmortem studies, control groups for comparison to cohorts with neurodegenerative, neuropsychiatric or neurological disease usually include donors without any known brain disease, although they show a variety of severity and type of somatic disease, which may also affect brain state (e.g., Alkemade et al. 2012; ten Kulve et al. 2016). Furthermore, donors with brain disorders may also have concomitant somatic diseases and have received appropriate pharmacological treatment. Finally, the stress of dying as well as the cause of death may influence brain characteristics (Sandberg et al. 1956; Uete et al. 1970; Aygen et al. 1997; Erkut et al. 2003). Although it is not possible to assess the effects of these individual factors on research outcomes, it is good research practice to report these potential confounders.

### Postmortem delay

After the demise of the donor, the blood is subjected to the influence of gravity. This means that if a deceased person is in a supine position, the blood will move towards the back of the brain with subsequent deformation effects. When postmortem delays increase, blood will coagulate in the blood vessels, which will negatively affect fixation efficiency if perfusion fixation is applied and autolysis will occur. Autolysis is an enzymatic reaction that occurs postmortem and which leads to the liquefaction of brain tissue. It is, therefore, best for tissue quality for histology that brain fixation takes place as soon as possible after death. This is in direct competition with the optimal conditions for performing postmortem diffusion-weighted MRI. Formalin significantly decreases T1 and T2 relaxation times, and mean water diffusivity (Birkl et al. 2016; Roebroeck et al. 2019). Diffusion-weighted imaging is, therefore, best performed shortly after the demise of a donor, before tissue fixation, followed by tissue preservation and histological processing. Scanning unfixed tissue thus comes at the expense of an increased postmortem interval before immersion into formalin.

### Tissue fixation steps

After the inevitable blood stasis, and onset of tissue degradation after death, researcher interference introduces further differences between the in vivo and postmortem MRI characteristics. Although a subset of postmortem in situ MRI studies are performed in fresh, unfixed tissue, many research groups scan formalin-fixed tissue. This choice can be steered by the availability of archival tissue, limited scan time availability, convenience, or subsequent steps in the planned research. To halt tissue degradation, brains are usually fixated either through perfusion fixation with formalin, but more often immersion fixation in formalin. In perfusion fixation, the fixative is pumped through the vascular system. With immersion fixation, the brain is removed from the skull and placed in a receptacle filled with fixative. Commonly, the brain is autopsied, and the meninges are removed before fixation. This inevitably causes tissue deformation through the opening of the sulci, which needs to be accommodated in the co-registration to individual or standard MRI templates. Formalin stops tissue degradation through protein cross-linking, which strongly affects MRI characteristics, resulting in T1 and T2* shortening, and with prolonged fixation times formalin crystals may form (van Duijn et al. 2011). In addition, the contrast of specific structures on MRI may even be inverted (Kirilina et al. 2019).

The processing of brain tissue for histology leads to further and more severe tissue deformation which may complicate the spatial alignment of the findings with an MRI common space for direct reuse of the data, and comparison across modalities. Again, a direct within subject comparison across modalities would provide a handle on the variation induced by the tissue fixation, as well as that the multimodal approach can provide an intermediate registration space that can be used for the translation across modalities. The advantages for using an intermediate MRI step are that well-established approaches for spatial alignment can be reused for the co-alignment of the data to MRI standard templates, and that the MRI data can serve as an external reference for the 3D reconstructions, to correct for Z-shifts (Malandain et al. 2004; Pichat et al. 2018).

### Controlling for and reporting of clinicopathological factors

Many of the clinicopathological factors are not easily controlled for when designing a study. Different approaches can be adopted, when performing comparisons between disease and control cohorts in postmortem studies, researchers may attempt to match for age and sex, and if possible for fixation duration and postmortem delays (Hestiantoro and Swaab 2004; Alkemade et al. 2005). However, combined MRI and microscopy studies often report case studies only, and it remains unclear how and to what extent the individual effects have contributed to the research results. Although it is impossible to determine the individual effects of such factors on the study outcome, it represents good research practice to report the available information.

At the same time, it is important to realize that the available clinical information for a donor is often incomplete. Donors may have been registered many years before their demise, and have progressed to develop somatic, neurological, and/or neurodegenerative disease. Information on pharmacological treatment at the end stages of life can be unavailable or incomplete as well, contributing to uncertainties on the generalizability of the data to the in vivo situation. Although a lot of these pre- and perimortem factors may remain unknown, it is possible, and even the gold standard, to assess neuropathological alterations after death. E.g., Braak staging can be performed to map the accumulation of the Alzheimer proteins beta-amyloid, which produces diffuse plaques, and neurofibrillary tangles containing hyperphosphorylated tau proteins. We would like to note that pathological alterations are thought to occur decades before the initial clinical presentation of Alzheimer’s disease, and clinical and postmortem observations do not show a one to one mapping (Bateman et al. 2012; Bennett et al. 2018). When histological processing of the brain tissue is performed, it is a relatively small but important investment to reserve sections from a number of specific brain areas to determine the extent of the neuropathological alterations using available guidelines (Montine et al. 2012).

## Movement, pulsation, liquid air interfaces

Studying postmortem tissue does not only pose technical challenges, it also brings opportunities. Inherent challenges in in vivo MRI brain studies include physiological motion, such as cardiac, respiratory, and gastrointestinal motion, in addition to vascular pulsation, and blood and CSF flow. The resulting artifacts include blurring and ghosting in the images (Zaitsev et al. 2015). As a result, brain structures that are located in close proximity of the large vessels of the brain are more prone to imaging artifacts. Similar limitations are present in structures that are close to liquid–air interfaces. These challenges are automatically resolved, or can be remedied postmortem. Movement and pulsation come to a halt, and the liquid air interface can be manipulated through the packaging of the tissue in susceptibility matched or inert liquid media such as Fomblin or Fluorinert (Miller et al. 2011; Massey et al. 2012; Weiss et al. 2015; Iglesias et al. 2018; Roebroeck et al. 2019). In addition, postmortem specimens can be scanned over an extended period of time, allowing more extensive and robust MRI protocols including single line readout protocols and the acquisition of additional scan repetitions that can be used to increase data quality.

## Translating between in vivo and postmortem observations

Comparisons across modalities can be either quantitative or qualitative. Qualitative comparisons often entail the visual comparison of microscopy data to MRI results. Studies are comparatively easy to conduct and have clear clinical and scientific merit. These approaches allow a qualitative interpretation of MRI contrasts which are related to pathology (e.g., tumors or (micro) lesions). Such visual comparisons only require approximate alignment with the brain, and can be performed without detailed co-registrations, which makes this approach also feasible for studies using smaller tissue blocks with a limited number of available anatomical landmarks for registration purposes. Quantitative approaches can include the correlating of MRI measurements of cortical thickness to postmortem assessments of the same parameters (Trampel et al. 2017; Wagstyl et al. 2018). These do not necessarily require an exact location match between the sites of measurement for being informative either. However, for quantification of image distortions on MRI, or for atlas creation, accurate spatial alignment between histology and MRI becomes a crucial factor. Full reconstructions can be successful, either with or without a blockface approach (Amunts et al. 2013; Alegro et al. 2016; Alho et al. 2018; Alkemade et al. 2022). An advantage of incorporating a blockface approach is the reduction of the registration process of the individual brain sections from a 2D-3D to a 2D-2D problem. This approach also prevents the complications posed by Z-shifts that can occur when a 2D to 3D approach is adopted without the use of a shape prior (Malandain et al. 2004). It is important to note that histological processing an entire human brain is a major feat, which requires both time and financial dedication. Importantly, whole-brain specimens are not readily available, and many researcher groups perform excellent work using tissue blocks. The feasibility of performing co-registration of tissue blocks in MRI templates is highly dependent on the anatomical information captured in these tissue blocks and can be based on landmarks.

Selected landmarks are required to be visible in both the tissue specimen and the target image. This is more likely to be successful if MRI contrasts are available from the same specimens, given the availability of a shape prior, as well as the anatomical characteristics visible in both the tissue block and the target template. Importantly, the registration results are highly dependent on the available anatomical landmarks present in the tissue block, and equally important, in the MRI target space. Furthermore, tissue blocks are often embedded in paraffin, after dehydration, which causes major (nonlinear) shape alterations. Histological sections are subsequently cut usually at a < 10 µm thickness, at which thickness they are prone to tearing as a result of burrs on the cutting knife, or the subsequent manipulation of the sections. More severe damage of sections requires labor-intensive manual repair of tissue artifacts.

An additional challenge is posed by the evaluation of the accuracy of the transformations. The accuracy of transformations can for instance be evaluated through the relative overlap of region labels available in both the tissue block and target template, and the alignment of anatomical fiducials (Paquola et al. 2021). One approach is to evaluate the registration result of structure that is under evaluation. Unfortunately, this then usually represents the evaluation of the registration of a landmark that was used for that same registration. As a result, this no longer represents an independent quality measure.

## Selecting a target space

Registration of microscopy data and MRI images in a common space creates a platform that allows interpretation of anatomical data across modalities. Multiple template spaces are available. The most established spaces are those released by the Montreal Neurological Institute (MNI spaces), which have underdone updating and continuous improvements over the years (Fonov et al. 2011). MNI templates play a prominent in the field of MRI, allowing co-registration of many types of acquired data (Evans et al. 2012; Xiao et al. 2019). Research groups performing postmortem MRI studies also consider these templates for registration of their results. However, postmortem MRI studies could potentially display more detailed anatomical contrast on which researchers would like to capitalize to improve registration algorithms and results. This creates a call for a novel postmortem reference space with a higher level of available anatomical contrast. In addition, the creation of a novel postmortem reference template allows the creation of multimodal incremental living atlases. The first presented whole-brain reconstruction BigBrain has now been included in Julich-Brain, which is a 3D probabilistic atlas of the cytoarchitecture of the human brain. For the BigBrain template, a toolbox has been created to map between the template and common MRI templates (Paquola et al. 2021). Such a toolbox is not needed if (ultra-high field quantitative) MRI and histological reconstructions are available within a single specimen. The MRI data then serve as a rich source of information, in itself, in addition to serving as a vehicle to allow the registration of the template to common MRI templates, such as the MNI template, using established toolboxes developed for and extensively used in MRI (e.g., ANTS, SPM, FSL, Freesurfer, MNE, AFNI, Slicer, Nipy; Cox 1996; Friston et al. 2007; Woolrich et al. 2009; Avants et al. 2011; Amunts Enkinson et al. 2012; Fedorov et al. 2012; Gramfort et al. 2013, 2014). It is important to co-align histological reconstructions to standard templates. Quality measures such as the calculation of Dice factors with region labels will then become possible as well as the incorporation of a probabilistic component (Dice and Dice 1945). This holds as well for individual in vivo data, however, direct registration of histological data is further complicated by the differences in the mutual information shared across modalities, which is used for registration purposes.

## Future outlooks and the development of incremental datasets

The human brain can be studied using in vivo or postmortem approaches. In vivo approaches allow the comparison across a large number of specimens, but are limited in their spatial resolution, number of repetitions, and movement artifacts. At the same time, postmortem studies provide unmatched anatomical detail, but allow only the histological processing of a limited number of samples. Individually, these approaches have limitations, combined they complement each other, providing the best of both worlds, and creating a powerful tool to improve our understanding of the human brain.

We would like to share a number of recommendations for postmortem studies that facilitate integration of research protocols: (1) ensure the availability of mutual information between imaging modalities for registration purposes. A straightforward approach is to perform histological staining that allows visualization of fiber bundles. This can be achieved using classical Luxol or Bielschowsky stainings. The derived contrast can be used for alignment with T1-derived MR contrasts. (2) Perform blockface imaging during the cutting process. The blockface reconstruction provides an important shape prior which is invaluable for preventing Z-shift effects and for reducing the image reconstruction from a 2D–3D to a 2D–2D problem. (3) Adapt tissue fixation to accommodate both MRI and histology. Formalin cross-linking prevents further tissue degradation at the expense of the MRI contrast. Consider performing (part of) the MRI imaging prior to fixation if possible, alternatively consider limiting formalin fixation and rinsing tissues extensively in buffered saline to partially recover shortened MRI decay times. (4) Consider compromising on the level of histological detail, to allow the publication together with state-of-the-art MRI data. Postmortem histological studies are very labor intensive and time consuming and represent a scientific tour de force. To decrease chances of MRI data no longer being state-of-the-art when histological processing is completed, compromises in acquiring histological detail can be considered. We would like to stress, however, that datasets with older MRI acquisitions should not be considered outdated, and still represent high scientific value. 5) Consider the prerequisites for optimizing the reusability of the data, particularly the registration to MRI templates. The applicability and reusability of the data is increased if the brain can be compared directly to in vivo brains, and more even if the data can be used to co-register annotations that cannot be made on in vivo MRI scans with lower anatomical detail and image contrast. To conclude, it is also important to keep in mind that template space is also subject to updating and continuous improvement. It is, therefore, important to not only share any created dataset in standard reference space, but also in individual template space, to allow also researchers developing new registration tools to make optimal use of the created datasets moving the field further forward.

## Data Availability

This declaration is not applicable.
